# Optimizing the use of biological therapy in patients with inflammatory bowel disease

**DOI:** 10.1093/gastro/gou087

**Published:** 2015-01-06

**Authors:** Alan C. Moss

**Affiliations:** Division of Gastroenterology, Beth Israel Deaconess Medical Center & Harvard Medical School, Boston, MA, USA

**Keywords:** inflammatory bowel disease, biological therapy

## Abstract

Biological therapy revolutionized the treatment of inflammatory bowel disease (IBD) during the last decade. These monoclonal antibodies, which target tumor necrosis factor (TNF), integrins or IL12/23, have been approved—or are in development for—both Crohn’s disease (CD) and ulcerative colitis (UC). Early use of these agents taught clinicians that induction and maintenance therapy, coupled with immunomodulator agents, reduced the immunogenicity of these agents, and led to sustained remission in many patients. More recent data has demonstrated that, through dose adjustments, optimizing serum drug levels may also provide more durable maintenance of remission, and improved mucosal healing. This review examines clinical practices that may enhance clinical outcomes from biological therapy in IBD.

## Introduction

Biological therapy currently refers to monoclonal antibodies directed against specific targets implicated in the pathogenesis of chronic inflammatory conditions. For inflammatory bowel disease (IBD), this primarily encompasses the approved anti-tumor necrosis factor (TNF) therapies (infliximab, adalimumab, certolizumab, golimumab), but also agents approved, or under development, that target integrins (natalizumab, vedolizumab) and interleukin (IL)-12/23 (ustekinumab), amongst others. Table 1 summarizes some of the biological therapies that have been studied in clinical trials in IBD.

**Table 1.** Examples of biologics for inflammatory bowel disease, approved and under development

Name	Primary target	FDA-approved indication
Infliximab (Remicade®)	TNF	CD, UC
Adalimumab (Humira®)	TNF	CD, UC
Certolizumab (Cimzia®)	TNF	CD
Golimumab (Simponi®)	TNF	UC
Natalizumab (Tysabri®)	alpha-4 integrin	CD
Vedolizumab (Entyvio®)	alpha-4-beta-7 integrin	CD, UC
Ustekinumab (Stelara®)	IL-12/23	N/A
Etrolizumab	beta-7 integrin	N/A
Anrukinzumab	IL-13	N/A

FDA = Food and Drug Administration; TNF = tumor necrosis factor; IL = interleukin; CD = Crohn’s disease; UC = ulcerative colitis; N/A = not available

The introduction of anti-TNF agents in the United States and Europe in the late 1990s gradually led to a paradigm shift in our approach to IBD therapy [1]. The deluge of studies that followed their approval provided evidence that this class of drug could induce sustained clinical remission in a cohort of patients, avoid the chronic need for steroids, reduce hospitalizations, and potentially prevent surgical interventions for complications [2]. The goal of treatment shifted from simply improving patients’ symptoms, to aiming for objective reversal of mucosal inflammation and prevention of long-term complications. On the down side, the use of anti-TNFs led in practice to reports of hypersensitivity reactions, reactivation of TB, and lymphomas in patients receiving these agents. The unprecedented enrolment of larger cohorts of IBD patients in registry studies to track adverse events led to a characterization of many disease- and drug- related safety signals [3]. For the first time, extensive capture of health outcomes, pharmaco-economic analyses, quality-of-life measures and work productivity were undertaken to assess the impact of these diseases—and their treatment—on the population of patients with IBD.

One of the conclusions of long-term follow-up studies, in practice and clinical trials, was that many patients who initially obtained ‘remission' with anti-TNF agents subsequently experienced reduced response over time: for example, in clinical trials, only ∼40% of patients were in remission at 1 year after induction, and in clinical practice less than 50% of initial responders were in remission at this time-point [4]. A review of these long-term outcomes suggests that there is clearly a population of patients who clinically respond to induction therapy and then maintain remission over time, with little need for dose adjustments; however, there is also a sizeable population of patients whose symptoms recur despite continued biological therapy. Strategies to address this problem and improve the rates of retention for this drug class have emerged in recent years [5].

## Mechanisms for loss of response to Biologics

The recurrence of clinical symptoms after induced remission with anti-TNFs is a common one; up to 60% of patients experience recurrence of symptoms in clinical practice over time [6]. There are many reasons for this event, and these are summarized in Table 2. The relative contribution of each cause is difficult to determine in practice, but cohort studies suggest that immunogenicity and overlap functional symptoms account for most cases of loss of response [7].

**Table 2.** Reasons for loss of clinical response amongst patients receiving biologics for inflammatory bowel disease

Mechanism
Immunogenicity (anti-drug antibodies)
Enhanced drug clearance (non-immunogenic)
Alternate inflammatory pathways
Non-inflammatory complications (e.g. strictures)
Overlap functional symptoms
Concurrent infections (e.g. *C. difficile* or Cytomegalovirus)

### Immunogenicity

Immunogenicity—the propensity for patients to develop anti-drug antibodies (ADAs) against the monoclonal agents—develops in a proportion of patients through both thyroid-dependent (high affinity, immunological memory), and thyroid independent (low affinity, occasional memory) mechanisms [8]. These ADAs are typically IgG antibodies that can impair binding of the biological agent to the target cytokine, or accelerate drug clearance by the reticulo-endothelial system (RES) [9]. When the anti-TNF antibody infliximab was initially given only intermittently in practice, up to 60% of patients developed anti-drug antibodies, and these patients were twice as likely as antibody-negative recipients to develop acute infusion reactions [10]. Since then, the use of induction and regular maintenance infusions has lowered the prevalence of anti-infliximab antibodies to 10–20% in randomized, controlled trials (RCTs) and observational cohorts [11]; in this patient population however, once they develop, ADAs are associated with a higher risk of loss of clinical response, and risk of infusions reactions [12, 13]. It has also recently been recognized that patients can develop transient ADAs, or persistent ADAs, and that drug-free periods lead to gradual disappearance of ADAs from circulation [14, 15]. While the factors that lead some patients to develop ADAs is only partially understood, persistently low drug serum levels have been associated with development of ADAs in some studies [16].

### Overlap functional symptoms

More frequent—but certainly less well-characterized—is the phenomenon of patients with IBD experiencing non-inflammatory symptoms. These are usually described as intestinal symptoms of pain, diarrhea or rectal bleeding in the absence of objective evidence of active inflammation on endoscopy. For example, in one study, 26% of patients with inactive Crohn’s disease met criteria for a diagnosis of irritable bowel syndrome (IBS) while, in another study, 10% of patients with quiescent ulcerative colitis (UC) had significant on-going abdominal pain [17, 18]. In some individuals, physiological processes such as delayed gastric emptying or bile salt malabsorption may play a role even in the absence of macroscopic inflammation [19, 20]. These factors need to be considered when evaluating recurrence of symptoms, as biological therapy for is not an appropriate intervention for IBS.

### Other processes

Whilst IBD manifests itself as a purely inflammatory process early on, later complications, such as strictures, fistulae, and malignancy, may lead to the development of symptoms that are unresponsive to any biological anti-cytokine therapy [9]. Small bowel strictures and their associated bacterial overgrowth can contribute to abdominal pain, bloating and diarrhea, and require mechanical correction [18]. Enteric fistulae to other abdominal compartments and organs may also lead to symptoms and a perceived loss of response to biological therapy.

## Strategies for optimizing the use of Biological therapy

The observations noted above illustrate that maintaining a sustained clinical remission with biological therapies requires attention to the many factors that could lead to relapse of symptoms. In the scenario where active inflammation has been objectively confirmed, and concurrent infections have been excluded, there are a number of therapeutic decisions that can be made to pro-actively, or reactively, enhance the efficacy of biological agents (Table 3). The evidence base for this is primarily derived from studies of infliximab, particularly with regard to serum drug levels and ADAs.

**Table 3.** Proposed strategies to optimize the efficacy of biological therapies in inflammatory bowel disease

Strategy	Evidence^a^
Patient selection	
Early in disease course	B
Administration schedule	
Induction & maintenance	B
Concomitant therapy	
Thiopurines	B
Methotrexate	C
Therapeutic drug monitoring	
Reactive testing	B
Proactive testing	C
Biomarker monitoring	
Proactive CRP measurements	C

^a^Grading of Recommendations Assessment, Development and Evaluation (GRADE) Working Group 2007.

A = several high-quality studies; B = moderate quality: several studies with limitations; C = low quality: studies with many limitations; CRP = C-reactive protein

### Patient selection

Biological agents were initially given only to patients with severe disease, which limited the potential long-term efficacy of these drugs. In RCTs of infliximab in Crohn’s disease, for example, enrolled subjects had the disease for an average of 8 years, 51% already required surgery for complications, and a third had failed to respond to thiopurines [21]. Since Crohn’s disease is a progressive condition, selecting only patients with long-standing disease for a given therapy reduces the proportion of recipients whose symptoms are caused primarily by active inflammation [22]. In contrast, *post-hoc* analysis of these RCTs has concluded that overall remission rates with biologics are numerically greater when administered to patients within 2 years of diagnosis [23]. Therefore, the preferred candidate for biological therapy is one with confirmed active intestinal inflammation, prior to the development of complications such as strictures. Using biologics in patients with overlap IBS or mostly fibrotic disease yields lower clinical remission rates, at considerable expense to those paying for treatment [24]. Of course, not every patient with IBD will require a biological therapy early after diagnosis. Selecting patients early in the course of their disease, who are at higher risk of complications, can be used to ‘risk stratify' individuals for early use of biological therapy. Clinical (age, phenotype, steroid use) and serogenetic factors have been identified, which are associated with relative risk of complications in Crohn’s disease, but the relative impact of biologics on low- or high-risk patients has yet to be prospectively determined [25].

### Maintenance schedules

The maintenance regimens tested in RCTs have confirmed that continued use of biologics is required to maintain remission in responsive patients. Episodic therapy leads, in practice, to higher rates of ADAs and infusion reactions, and lower rates of remission [10, 26]. Patients with Crohn’s disease receiving scheduled infliximab had lower disease activity scores and fewer hospitalizations, but higher response rates, than patients who received episodic therapy [26]. The recommended schedule for administration (every other week, every 4 weeks or every 8 weeks) may in some cases be insufficient to maintain remission, and schedules of administration that are more frequent than the approved dose are commonly used [6, 27]. A number of cohort studies have reported that these ‘escalation strategies' are able to re-capture response in up to 80% of patients who have lost response [28, 29].

### Concurrent immunomodulators

In some studies, the administration of concurrent immunomodulators (IMMs), such as azathioprine or methotrexate, has been associated with higher remission rates and lower rates of ADAs. *Post-hoc* analysis of the RCTs of anti-TNFs, and retrospective review of clinical cohorts, did not demonstrate superior efficacy of combination therapy over monotherapy in Crohn’s disease [30, 31]; however, subsequent prospective trials in both CD and UC concluded that initial combination therapy was beneficial. In the SONIC (Study of Biologic and Immunomodulator Naive Patients in Crohn’s Disease) trial in CD, 57% of patients receiving infliximab and thiopurines were in corticosteroid-free clinical remission at week 26, as compared with 44% of those receiving infliximab alone (*P = *0.02) [32]. Similarly, in the SUCCESS (Efficacy and Safety of Infliximab, as Monotherapy or in Combination with Azathioprine, versus Azathioprine Monotherapy in Moderate to Severe Ulcerative Colitis) trial in UC, steroid-free remission was achieved by 40% of patients receiving infliximab or azathioprine, compared with 22% receiving infliximab alone (*P = *0.017) [33]. The reason for the synergistic effects of immunomodulators may be their ability to reduce the immunogenicity of biologics. In the SONIC trial, ADAs developed in less than 1% of patients receiving azathioprine and infliximab, in contrast to 15% of patients who received infliximab alone [32]. In one small case series, even *post-hoc* addition of an immunomodulator to a biologic could reduce ADAs, and elevate serum drug levels, in patients with CD [34].

### Therapeutic drug monitoring

Many observational studies have linked low serum drug levels to a higher risk of ADA development, and/or loss of response to biologics in IBD [35–37]. In response, reactive measurement of ADAs and serum drug levels using Enzyme Linked Immunosorbent Assay (ELISA) assays, and appropriate adjustment of drug regimen, has been utilized in practice to optimize clinical outcomes [28, 38]. This strategy has been reported to be more cost-effective than empirical escalation of doses of biologics, with similar clinical outcomes in this setting [7, 39]. In Denmark, Steenholdt *et al*. concluded that individualized infliximab therapy, based on drug levels, was more cost-effective than empirical dose intensification in patients losing response to infliximab; response rates were similar (∼55%), but costs were 34% lower when therapeutic drug monitoring was used [7].

In the light of these findings, other groups have examined proactive adjustment of biological drug levels to prevent relapse of disease. The Trough level Adapted infliXImab Treatment (TAXIT) trial from Europe enrolled patients in remission and adjusted their infliximab dose to obtain a target serum drug level; subsequently this cohort was randomized to either standard care, or continued adjustment of infliximab dose, based on drug levels. After 1 year, overall remission rates were similar in both arms (69% and 72%) [40]. A small retrospective analysis from another group reported that patients who had proactive adjustment of infliximab levels to above 5 µg/mL were less likely to discontinue infliximab than those who were not adjusted (10% *vs.* 31%, respectively; *P = *0.009) [41]. Where commercial assays for biological drug levels are not available, C-reactive protein (CRP) and fecal calprotectin may be surrogate markers to identify patients at risk of relapse due to low drug levels [42–44]. One area of uncertainty is the drug level to aim for when using therapeutic monitoring; many studies have used different cut-offs, so it is unclear what is the ideal therapeutic range (Table 4) [16, 35, 45–54].

**Table 4.** Drug thresholds used to categorize patients’ outcomes in observational studies

Study	Drug	Threshold (μg/mL)	Outcome
Ben-Bassat O [35]	IFX	2	Remission
Drastich P [45]	IFX	2	Remission
Murthy S [46]	IFX	2	Remission
Maser EA [47]	IFX	1.4	Remission
Reinisch W [48]	IFX	3	Remission
Seow CH [49]	IFX	2	Remission
Steenholdt C [50]	IFX	0.5	Remission
Vande Casteele [16]	IFX	2.2	CRP
Feagan BG [51]	IFX	3	CRP
Bortlik M [52]	IFX	3	CRP
Mazor Y [53]	ADA	5	Remission
Imaeda H [54]	ADA	10	CRP

IFX = infliximab; ADA = adalimumab; CRP = C-reactive protein

## Conclusions

**Figure 1.**
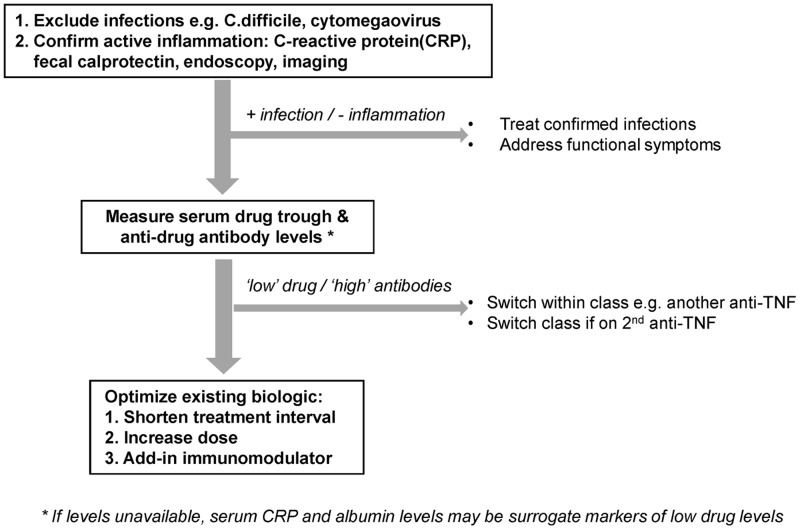
Strategies of biologics use in patients with inflammatory bowel disease

There has been a steep learning curve in the optimal use of biologics in IBD since 1997. The current strategies of patient selection, maintenance schedules, use of concurrent immunomodulators, and therapeutic drug monitoring have generated incremental improvements in the long-term remission rates with this class of drug. We are still not at the point where the majority of patients treated with biologics achieve sustained clinical and/or endoscopic remission. Alterations in immunological pathways over time, persistence of functional intestinal symptoms, and differences in underlying pathogenic processes may prevent this being universally achievable; however, optimal use of biologics in those who initially respond to them will certainly enhance the efficacy of this drug class in the medium term (Figure 1).

## Funding

ACM is supported by NIH grant K23DK084338.

*Conflict of interest statement*. ACM has consulted for Janssen, Abbott, UCB, Roche and Bayer, and received research support (to BIDMC) from Pfizer.

## References

[1] Antunes O Filippi J Hebuterne X Treatment algorithms in Crohn's: up, down or something else? Best Pract Res Clin Gastroenterol 2014;28:473–83. 24913386 10.1016/j.bpg.2014.05.001

[2] Mandel MD Miheller P Mullner K Have biologics changed the natural history of Crohn's disease? Dig Dis 2014;32:351–9. 24969279 10.1159/000358135

[3] Lichtenstein GR Feagan BG Cohen RD Drug therapies and the risk of malignancy in Crohn's disease: results from the TREAT Registry. Am J Gastroenterol 2014;109:212–23. 24394749 10.1038/ajg.2013.441

[4] Gisbert JP Panes J Loss of response and requirement of infliximab dose intensification in Crohn's disease: a review. Am J Gastroenterol 2009;104:760–7. 19174781 10.1038/ajg.2008.88

[5] Vande Casteele N Feagan BG Gils A Therapeutic drug monitoring in inflammatory bowel disease: current state and future perspectives. Curr Gastroenterol Rep 2014;16:378. 24595615 10.1007/s11894-014-0378-0

[6] Karmiris K Paintaud G Noman M Influence of trough serum levels and immunogenicity on long-term outcome of adalimumab therapy in Crohn's disease. Gastroenterology 2009;137:1628–40. 19664627 10.1053/j.gastro.2009.07.062

[7] Steenholdt C Brynskov J Thomsen OO Individualised therapy is more cost-effective than dose intensification in patients with Crohn's disease who lose response to anti-TNF treatment: a randomised, controlled trial. Gut 2014;63:919–27. 23878167 10.1136/gutjnl-2013-305279

[8] Sauerborn M Brinks V Jiskoot W Immunological mechanism underlying the immune response to recombinant human protein therapeutics. Trends Pharmacol Sci 2010;31:53–9. 19963283 10.1016/j.tips.2009.11.001

[9] Fasanmade AA Adedokun OJ Blank M Pharmacokinetic properties of infliximab in children and adults with Crohn's disease: a retrospective analysis of data from two phase III clinical trials. Clin Ther 2011;33:946–64. 21741088 10.1016/j.clinthera.2011.06.002

[10] Baert F Noman M Vermeire S Influence of immunogenicity on the long-term efficacy of infliximab in Crohn's disease. N Engl J Med 2003;348:601–8. 12584368 10.1056/NEJMoa020888

[11] Hanauer SB Wagner CL Bala M Incidence and importance of antibody responses to infliximab after maintenance or episodic treatment in Crohn's disease. Clin Gastroenterol Hepatol 2004;2:542–53. 15224278 10.1016/s1542-3565(04)00238-1

[12] O'Meara S Nanda KS Moss AC Antibodies to infliximab and risk of infusion reactions in patients with inflammatory bowel disease: a systematic review and meta-analysis. Inflamm Bowel Dis 2014;20:1–6. 24280879 10.1097/01.MIB.0000436951.80898.6d

[13] Nanda KS Cheifetz AS Moss AC Impact of antibodies to infliximab on clinical outcomes and serum infliximab levels in patients with inflammatory bowel disease (IBD): a meta-analysis. Am J Gastroenterol 2013;108:40–7; quiz 48. 23147525 10.1038/ajg.2012.363 PMC3561464

[14] Ben Horin S Mazor Y Yanai H The decline of anti-drug antibody titres after discontinuation of anti-TNFs: implications for predicting re-induction outcome in IBD. Aliment Pharmacol Ther 2012;35:714–22. 22288419 10.1111/j.1365-2036.2012.04997.x

[15] van de Casteele N Cuypers L Singh S Antibodies to Infliximab can either be persistent or transient; a retrospective case-control study in IBD patients treated with Infliximab maintenance therapy. Gastroenterology 2012;143:563.

[16] Vande CN Gils A Singh S Antibody response to infliximab and its impact on pharmacokinetics can be transient. Am J Gastroenterol 2013;108:962–71. 23419382 10.1038/ajg.2013.12

[17] Coates MD Lahoti M Binion DG Abdominal pain in ulcerative colitis. Inflamm Bowel Dis 2013;19:2207–14. 23929261 10.1097/MIB.0b013e31829614c6 PMC3749243

[18] Farrokhyar F Marshall JK Easterbrook B Functional gastrointestinal disorders and mood disorders in patients with inactive inflammatory bowel disease: prevalence and impact on health. Inflamm Bowel Dis 2006;12:38–46. 16374257 10.1097/01.mib.0000195391.49762.89

[19] Gothe F Beigel F Rust C Bile acid malabsorption assessed by 7 alpha-hydroxy-4-cholesten-3-one in pediatric inflammatory bowel disease: correlation to clinical and laboratory findings. J Crohns Colitis 2014;8:1072–8. 24666974 10.1016/j.crohns.2014.02.027

[20] Nobrega AC Ferreira BR Oliveira GJ Dyspeptic symptoms and delayed gastric emptying of solids in patients with inactive Crohn's disease. BMC Gastroenterol 2012;12:175. 23216812 10.1186/1471-230X-12-175 PMC3537636

[21] Hanauer SB Feagan BG Lichtenstein GR Maintenance infliximab for Crohn's disease: the ACCENT I randomised trial. Lancet 2002;359:1541–9. 12047962 10.1016/S0140-6736(02)08512-4

[22] Cosnes J Gower-Rousseau C Seksik P Epidemiology and natural history of inflammatory bowel diseases. Gastroenterology 2011;140:1785–94. 21530745 10.1053/j.gastro.2011.01.055

[23] Cornillie F Hanauer S Diamond RH Can clinical, biological or pharmacological markers predict sustained response to infliximab? A retrospective analysis of ACCENT 1. Gut 2011;60:A296. 10.1136/gutjnl-2012-304094 PMC4215276 24474383

[24] Liu Y Wu EQ Bensimon AG Cost per responder associated with biologic therapies for Crohn's disease, psoriasis, and rheumatoid arthritis. Adv Ther 2012;29:620–34. 22843208 10.1007/s12325-012-0035-7

[25] Beaugerie L Sokol H Clinical, serological and genetic predictors of inflammatory bowel disease course. World J Gastroenterol 2012;18:3806–13. 22876031 10.3748/wjg.v18.i29.3806 PMC3413051

[26] Rutgeerts P Feagan BG Lichtenstein GR Comparison of scheduled and episodic treatment strategies of infliximab in Crohn's disease. Gastroenterology 2004;126:402–13. 14762776 10.1053/j.gastro.2003.11.014

[27] Rostholder E Ahmed A Cheifetz AS Outcomes after escalation of infliximab therapy in ambulatory patients with moderately active ulcerative colitis. Aliment Pharmacol Ther 2012;35:562–7. 22239070 10.1111/j.1365-2036.2011.04986.x PMC3277945

[28] Afif W Loftus EV Jr Faubion WA Clinical utility of measuring infliximab and human anti-chimeric antibody concentrations in patients with inflammatory bowel disease. Am J Gastroenterol 2010;105:1133–9. 20145610 10.1038/ajg.2010.9 PMC6937708

[29] Billioud V Sandborn WJ Peyrin-Biroulet L Loss of response and need for adalimumab dose intensification in Crohn's disease: a systematic review. Am J Gastroenterol 2011;106:674–84. 21407178 10.1038/ajg.2011.60

[30] Lichtenstein GR Diamond RH Wagner CL Clinical trial: benefits and risks of immunomodulators and maintenance infliximab for IBD-subgroup analyses across four randomized trials. Aliment Pharmacol Ther 2009;30:210–26. 19392858 10.1111/j.1365-2036.2009.04027.x

[31] Moss AC Kim KJ Fernandez-Becker N Impact of concomitant immunomodulator use on long-term outcomes in patients receiving scheduled maintenance infliximab. Dig Dis Sci 2010;55:1413–20. 19533357 10.1007/s10620-009-0856-7

[32] Colombel JF Sandborn WJ Reinisch W Infliximab, azathioprine, or combination therapy for Crohn's disease. N Engl J Med 2010;362:1383–95. 20393175 10.1056/NEJMoa0904492

[33] Panaccione R Ghosh S Middleton S Combination therapy with infliximab and azathioprine is superior to monotherapy with either agent in ulcerative colitis. Gastroenterology 2014;146:392–400 e3. 24512909 10.1053/j.gastro.2013.10.052

[34] Ben Horin S Waterman M Kopylov U Addition of an immunomodulator to infliximab therapy eliminates antidrug antibodies in serum and restores clinical response of patients with inflammatory bowel disease. Clin Gastroenterol Hepatol 2013;11:444–47. 23103905 10.1016/j.cgh.2012.10.020

[35] Ben-Bassat O Romanova A Lacono A Association of serum infliximab and antibodies to infliximab to long-term clinical outcome and mucosal healing in Crohn's disease. Gastroenterology 2013;144 (5 Suppl. 1):S775.

[36] Cesarini M Katsanos K Papamichael K Dose optimization is effective in ulcerative colitis patients losing response to infliximab: a collaborative multicentre retrospective study. Dig Liver Dis 2014;46:135–9. 24246151 10.1016/j.dld.2013.10.007

[37] Colombel JF Sandborn WJ Allez M Association Between Plasma Concentrations of Certolizumab Pegol and Endoscopic Outcomes of Patients with Crohn's Disease. Clin Gastroenterol Hepatol 2014;12:423–31 e1. 24184736 10.1016/j.cgh.2013.10.025

[38] Vande Casteele N Ballet V Van Assche G Early serial trough and antidrug antibody level measurements predict clinical outcome of infliximab and adalimumab treatment. Gut 2012;61:321. 21330576 10.1136/gut.2010.236869

[39] Velayos FS Kahn JG Sandborn WJ A test-based strategy is more cost effective than empiric dose escalation for patients with Crohn's disease who lose responsiveness to infliximab. Clin Gastroenterol Hepatol 2013;11:654–66. 23357488 10.1016/j.cgh.2012.12.035

[40] vande Casteele N Gils A Ballet V Randomised controlled trial of drug level versus clinically based dosing of infliximab maintenance therapy in IBD: final results of the TAXIT study. United European Gastroenterol J 2013;1:A1–134.

[41] Vaughn BP Martinez-Vazquez M Patwardhan VR Proactive therapeutic concentration monitoring of infliximab may improve outcomes for patients with inflammatory bowel disease: results from a pilot observational study. Inflamm Bowel Dis 2014;20:1996–2003. 25192499 10.1097/MIB.0000000000000156 PMC5557346

[42] Peyrin-Biroulet L Reinisch W Colombel JF Clinical disease activity, C-reactive protein normalisation and mucosal healing in Crohn's disease in the SONIC trial. Gut 2014;63:88–95. 23974954 10.1136/gutjnl-2013-304984

[43] Velayos F Sheibani S Lockton S Prevalence of antibodies to adalimumab (ATA) and correlation between ATA and low serum drug concentration on CRP and clinical symptoms in a prospective sample of IBD patients. Gastroenterology 2013;144 (5 Suppl. 1):S490.

[44] Reinisch W Colombel JF Sandborn WJ Infliximab serum trough level and CRP change are associated with corticosteroid-free remission in Crohn's disease: a *post-hoc* analysis of the sonic trial. Gut 2012;61 (Suppl. 2):A170.

[45] Drastich P Kozeluhova J Jaresova M Infliximab serum trough levels and deep remission in patients with IBD. Gastroenterology 2011;140 (5 Suppl. 1):S292.

[46] Murthy S Kevans D Seow CH Association of serum infliximab and antibodies to infliximab to long-term clinical outcome in acute ulcerative colitis. Gastroenterology 2012;142 (5 Suppl. 1):S388.

[47] Maser EA Villela R Silverberg MS Association of trough serum infliximab to clinical outcome after scheduled maintenance treatment for Crohn's disease. Clin Gastroenterol Hepatol 2006;4:1248–54. 16931170 10.1016/j.cgh.2006.06.025

[48] Reinisch W Feagan BG Rutgeerts PJ Infliximab concentration and clinical outcome in patients with ulcerative colitis. Gastroenterology 2012;142 (5 Suppl. 1):S114.

[49] Seow CH Newman A Irwin SP Trough serum infliximab: a predictive factor of clinical outcome for infliximab treatment in acute ulcerative colitis. Gut 2010;59:49–54. 19651627 10.1136/gut.2009.183095

[50] Steenholdt C Bendtzen K Brynskov J Cut-off levels and diagnostic accuracy of infliximab trough levels and anti-infliximab antibodies in Crohn's disease. *Scand J Gastroentero*l 2011;46:310–18. 21087119 10.3109/00365521.2010.536254

[51] Feagan BG Singh S Lockton S Novel infliximab (IFX) and antibody-to-infliximab (ATI) assays are predictive of disease activity in patients with Crohn's disease (CD). Gastroenterology 2012;142 (5 Suppl. 1):S114.

[52] Bortlik M Duricova D Malickova K Infliximab trough levels may predict sustained response to infliximab in patients with Crohn's disease. J Crohns Colitis 2013;7:736–43. 23200919 10.1016/j.crohns.2012.10.019

[53] Mazor Y Almog R Kopylov U Adalimumab drug and antibody levels as predictors of clinical and laboratory response in patients with Crohn's disease. Aliment Pharmacol Ther 2014;40:620–8. 25039584 10.1111/apt.12869

[54] Imaeda H Takahashi K Fujimoto T Accurate determination of serum adalimumab and anti-adalimumab antibodies levels during maintenance therapy for Crohn's disease. Gastroenterology 2013;144 (5 Suppl. 1):S431.

